# Amiloride Improves Endothelial Function and Reduces Vascular Stiffness in Female Mice Fed a Western Diet

**DOI:** 10.3389/fphys.2017.00456

**Published:** 2017-06-30

**Authors:** Luis A. Martinez-Lemus, Annayya R. Aroor, Francisco I. Ramirez-Perez, Guanghong Jia, Javad Habibi, Vincent G. DeMarco, Brady Barron, Adam Whaley-Connell, Ravi Nistala, James R. Sowers

**Affiliations:** ^1^Dalton Cardiovascular Research Center, University of MissouriColumbia, MO, United States; ^2^Department of Biological Engineering, University of MissouriColumbia, MO, United States; ^3^Department of Medical Pharmacology and Physiology, University of MissouriColumbia, MO, United States; ^4^Research Service, Harry S. Truman Memorial Veterans' HospitalColumbia, MO, United States; ^5^Diabetes and Cardiovascular Research Center, University of MissouriColumbia, MO, United States; ^6^Division of Nephrology and Hypertension, University of MissouriColumbia, MO, United States

**Keywords:** EnNaC inhibition, endothelial dysfunction, vascular remodeling, obesity, pulse wave velocity

## Abstract

Obese premenopausal women lose their sex related cardiovascular disease protection and develop greater arterial stiffening than age matched men. In female mice, we have shown that consumption of a Western diet (WD), high in fat and refined sugars, is associated with endothelial dysfunction and vascular stiffening, which occur via activation of mineralocorticoid receptors and associated increases in epithelial Na^+^ channel (ENaC) activity on endothelial cells (EnNaC). Herein our aim was to determine the effect that reducing EnNaC activity with a very-low-dose of amiloride would have on decreasing endothelial and arterial stiffness in young female mice consuming a WD. To this end, we fed female mice either a WD or control diet and treated them with or without a very-low-dose of the ENaC-inhibitor amiloride (1 mg/kg/day) in the drinking water for 20 weeks beginning at 4 weeks of age. Mice consuming a WD were heavier and had greater percent body fat, proteinuria, and aortic stiffness as assessed by pulse-wave velocity than those fed control diet. Treatment with amiloride did not affect body weight, body composition, blood pressure, urinary sodium excretion, or insulin sensitivity, but significantly reduced the development of endothelial and aortic stiffness, aortic fibrosis, aortic oxidative stress, and mesenteric resistance artery EnNaC abundance and proteinuria in WD-fed mice. Amiloride also improved endothelial-dependent vasodilatory responses in the resistance arteries of WD-fed mice. These results indicate that a very-low-dose of amiloride, not affecting blood pressure, is sufficient to improve endothelial function and reduce aortic stiffness in female mice fed a WD, and suggest that EnNaC-inhibition may be sufficient to ameliorate the pathological vascular stiffening effects of WD-induced obesity in females.

## Introduction

Stiffening of the arterial vasculature is highly predictive of increased cardiovascular disease (CVD) including macro and microvascular associated pathologies such as coronary artery disease, peripheral artery disease, and stroke, as well as the development of proteinuria and chronic kidney disease (CKD; Mitchell, 2009). It is thought that proteinuria in the presence of arterial stiffening increases the risk for CVD, and is a strong functional marker for arterial stiffening in obesity (Taal et al., 2007). This is particularly relevant in women, as it has been shown that female obesity and diabetes associated with consumption of a Western diet (WD) high in fat and refined sugars increases vascular stiffness (Harvey et al., 2016; Jia et al., 2016). Consequently, as the obesity epidemic expands, so does arterial stiffening and associated CVD and CKD. Moreover, in agreement with its characterization as a strong surrogate predictor, it is becoming clear that stiffening of the arterial circulation occurs prior to the development of other risk factors for CVD and CKD such as hypertension (Weisbrod et al., 2013). Therefore, elucidation of the mechanisms that control arterial stiffening, as well as identification of preventative and therapeutic approaches for reducing arterial stiffness have the potential of providing important avenues for ameliorating CVD and CKD, especially in obese and diabetic women.

Human population studies show that women with obesity and diabetes have a greater risk for CVD than comparable men (Natarajan et al., 2003; Mitchell, 2009; Regensteiner et al., 2015). This sex difference prevails in age adjusted data and pre-menopausal women, suggesting that obese women lose their sex-associated cardiovascular protection and develop CVD at a greater incidence than age-matched men (DeMarco et al., 2014). Furthermore, it has also been found that abdominal adiposity has a greater association with arterial stiffness in young women than in men (Scuteri et al., 2012), which again suggests that obesity increases the risk for CVD to a greater extent in pre-menopausal women than in men. We previously demonstrated that young female mice fed a WD have increased aortic pulse wave velocity (PWV), indicative of arterial stiffening (Bender et al., 2015; DeMarco et al., 2015; Jia et al., 2016). We also found that aortic PWV did not increase in WD-fed female mice lacking mineralocorticoid receptors (MRs) in endothelial cells (ECMR^−/−^) (Jia et al., 2016). This indicated that a major mechanism involved in WD-induced arterial stiffening involves enhanced MR signaling in the vascular endothelium. The protection from arterial stiffening conferred by ECMR^−/−^ was also associated with increased flow-induced vasodilation and vascular nitric oxide (NO) bioavailability, as well as with reductions in arterial fibrosis, oxidative stress, vascular wall hypertrophy, inflammation, presence of epithelial Na^+^ channels (ENaC) in the endothelium (EnNaC), endothelial stiffness and endothelial dysfunction. Our results, therefore, suggest that WD feeding induces arterial stiffening through a pathway that requires ECMR signaling and the subsequent augmented activity of EnNaC, endothelial stiffening, oxidative stress, and reduced NO bioavailability.

ENaC is a multimeric channel responsible for maintenance of cellular Na^+^, and consequently cell water and volume homeostasis. Its physiological role in Na^+^ transport has been well-described in the kidney, colon, lungs, and sweat glands (Garty and Palmer, 1997). Recently, ENaC has been shown to be present in vascular endothelium, where it is termed EnNaC (Warnock, 2013). There, its role in allowing Na^+^ entrance into cells is associated with endothelial cortical stiffening and a reduced capacity of the endothelium to activate endothelial NO synthase (eNOS) in response to insulin or shear stress (Oberleithner et al., 2007). In support for a pathway in which WD-consumption leads to vascular endothelial dysfunction via augmented EnNaC activity, we have shown that EnNaC inhibition with a very-low-dose of amiloride, improves endothelium-dependent vasodilatory responses to acetylcholine in aortae and to flow in resistance arteries (Jia et al., 2016). Furthermore, EnNaC inhibition with amiloride in cultured endothelial cells has been shown to reduce cortical stiffness in cells exposed to aldosterone (Oberleithner et al., 2006). Based on these results, we hypothesized that reducing the activity of EnNaC via very-low-dose amiloride administration would prevent the development of conduit and resistance artery stiffening in female mice fed a WD, and that this arterial de-stiffening would be accompanied by amelioration of the negative effects we have previously reported WD consumption has on arterial structure and function.

## Methods

### Animals

All animal procedures were performed in accordance with guidelines from the National Institutes of Health and pre-approved by animal care and use committees at the University of Missouri and the Harry S. Truman Veterans' Memorial Hospital. Black C57/6J female mice were purchased from Jackson Laboratories (Bar Harbor, ME), while ECMR^−/−^ female mice were generated as previously described (Jia et al., 2016). Animals were fed either a control diet (CD) or a WD (46% fat, 17.5% sucrose, and 17.5% high fructose corn syrup, Test Diet 58Y1, Richmond, Indiana) for 20 weeks beginning at 4 weeks of age. Half the mice on either diet received 1 mg/kg/day amiloride in the drinking water for the same 20-weeks. Euthanasia was achieved by pneumothorax followed by exsanguination in mice that were under surgical plane anesthesia induced by isoflurane inhalation.

### Body composition

Body composition was assessed using an EchoMRI-500 Analyzer (Echo Medical Systems, Houston, TX, USA) in 20-week old conscious mice as previously described (Jia et al., 2015a,b). Quantitative magnetic resonance analysis of whole body fat mass, lean mass and total body water was determined in all mice cohorts, which consisted of: (1) Mice fed CD and receiving no amiloride (CDC), (2) Mice fed CD and receiving amiloride (CDA), (3) Mice fed WD and receiving no amiloride (WDC), and (4) Mice fed WD and receiving amiloride (WDA). Body weights were obtained at 24 weeks of age just prior to euthanasia in animals that were fasted for 5 h. Blood samples were obtained after animals were weighed and plasma glucose determined with the use of a glucometer. Additional plasma parameters were measured by a commercial laboratory (Comparative Clinical Pathology Service, LLC Columbia, MO).

### Urine analyses

Urine samples were collected within 1 week prior to sacrifice from mice placed in metabolic cages for 24 h. Total protein content in the urine was determined using a Beckman Coulter colorimetric assay run on an automated clinical chemistry analyzer (AU680, Beckman-Coulter, Inc., Brea, CA; Nistala et al., 2014). Creatinine was measured by the Jaffe reaction using the same analyzer and used for the normalization of proteinuria. Creatinine was also measured using an enzymatic creatinine method and the 2 values were compared (Diazyme, Poway CA). Urine sodium was measured by the same Beckman-Coulter analyzer employing ion-specific electrodes.

### Blood pressure and aortic pulse wave velocity

Blood pressure was determined only in subsets of anesthetized WDC and WDA mice via carotid artery catheterization as previously described (Jia et al., 2015a). Calculation of PWV between the thoracic and abdominal aorta was performed using a Doppler ultrasound (Indus Mouse Doppler System, Webster, TX) as previously described (Bender et al., 2015; Jia et al., 2016). In order to control for the potential effects of heart rate, all time measurements were gated to a heart rate of 400 beats per minute. Blood pressure and PWV were determined within 1 week prior to euthanasia.

### *Ex vivo* vasomotor responses

At euthanasia, samples of mesenteric resistance arteries were collected for determination of vascular functional responses as we have previously described (Foote et al., 2016). Briefly, isolated mesenteric resistance arteries were cannulated and mounted in pressure myographs at 70 mmHg, preconstricted with 10^−5^ M phenylephrine and exposed to increasing concentrations of acetylcholine and sodium nitroprusside.

### Endothelial stiffness

The elastic modulus or stiffness of the aortic endothelial surface was measured in thoracic aorta explants using an atomic force microscope (AFM) MFP-3D AFM 89 (Asylum Research Inc. Goleta, CA) as previously described (Jia et al., 2016). Briefly, a segment of the thoracic aorta was opened longitudinally and glued on a glass slide using Cell-Tak. A protocol of nano-indentation and -retraction cycles was applied on the endothelial surface of samples kept at room temperature (~25°C) and the curves generated were used to calculate the elastic modulus (stiffness) of the cell surface (Jia et al., 2016). A subset of aortic explants from WDC-fed mice was exposed *ex vivo* to a solution containing amiloride (1 μM) or vehicle control for 4 h before the AFM nano-indentation protocol was performed.

### Vascular structure and remodeling characteristics

Segments of the thoracic aorta were fixed in 4% paraformaldehyde, and prepared for imaging as previously described (Jia et al., 2016). Picrosirius red was used to stain collagen and Verhoeff–Van Gieson staining was used to determine aortic wall thickness. Specific primary antibodies were used to detect nitrotyrosine (1:200 dilution of Dako 2017-07) and ENaC (1:200 dilution of Thermo Scientific PA1-920A). Corresponding secondary antibodies tagged with fluorescent probes were used to identify the proteins detected by the primary antibodies. Incubation in secondary antibody only was used as control. Isolated mesenteric arteries were fixed in 4% paraformaldehyde while pressurized intraluminally to 70 mmHg. Subsequently, the arteries were exposed to a primary antibody vs. ENaC (1:200 dilution of Thermo Scientific PA1-920A) followed by a secondary antibody tagged with Alexa 488 (1:1,000 dilution of Invitrogen A11034). Concomitant with the secondary antibody, arteries were incubated with 0.5 μg/ml 4,6-diamidino-2-phenylindole (DAPI) to stain nuclei, 0.2 μM Alexa Fluor 633 hydrazide (Molecular Probes) to stain elastin, and 0.02 μM Alexa Fluor 546 phalloidin (Molecular Probes) to stain F-actin, as previously described (Bender et al., 2015). Images detecting all fluorescent probes and collagen with second harmonic image generation within the mesenteric vessels were acquired using a Leica SP5 confocal/multiphoton microscope (Bender et al., 2015).

### Statistics

All data are presented as means ± SE. Statistical analyses consisted of *t*-tests or ANOVA with Bonferroni's correction for multiple comparisons where appropriate. A *p* ≤ 0.05 was considered significant.

## Results

### Effects of *in vivo* administration of very-low-dose amiloride on body composition, urine, and plasma analyses

**Figure 1:**
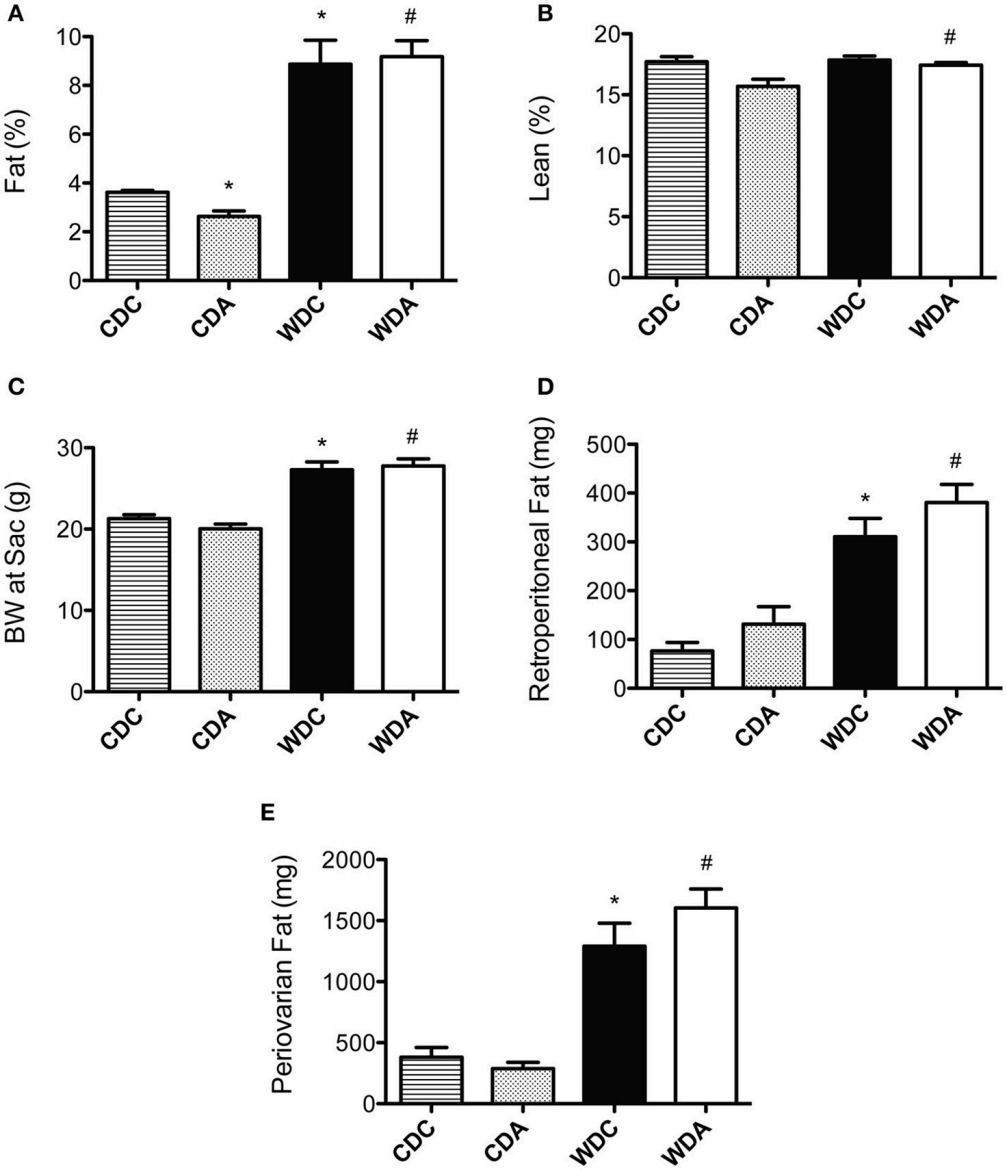
Effects of Western diet (WD) consumption and amiloride treatment on body composition. **(A)** Body fat percent in 20-week-old female mice fed either a control diet (CD) or a WD and treated (CDA, WDA) or not (CDC, WDC) with amiloride (1 mg/kg/day) in the drinking water. **(B)** Percent lean mass in the same mice cohorts. **(C)** Body weight at sacrifice (24-week-old) in the same mice cohorts. **(D)** Amount of retroperitoneal fat present at sacrifice in the same mice cohorts. **(E)** Amount of periovarian fat present at sacrifice in the same mice cohorts. Data are means ± SE of *n* = 4–13. ^*^P < 0.05 vs. CDC, ^#^vs. CDA.

Because changes in body composition have been linked to vascular remodeling processes (Strasser et al., 2015), we first determined whether amiloride administration affected body fat and lean mass in mice fed either diet. As previously reported (Jia et al., 2015b), WD consumption increased whole body fat mass as detected by echo-MRI analysis (Figure 1A). *In vivo* very-low-dose amiloride administration caused a small but significant reduction in fat content only in CD-fed animals, but not in those fed a WD (Figure 1A). Lean body mass was slightly but significantly increased in mice fed a WD and treated with amiloride compared to those treated with amiloride but fed a CD (Figure 1B). Body weight at sacrifice, as well as retroperitoneal and peri-ovarian fat, were greater in mice fed a WD, and not affected by amiloride (Figures 1C–E). These data indicate very-low-dose amiloride had only a small effect on fat content in mice fed a CD and no effect in the body composition of mice fed a WD.

**Figure 2:**
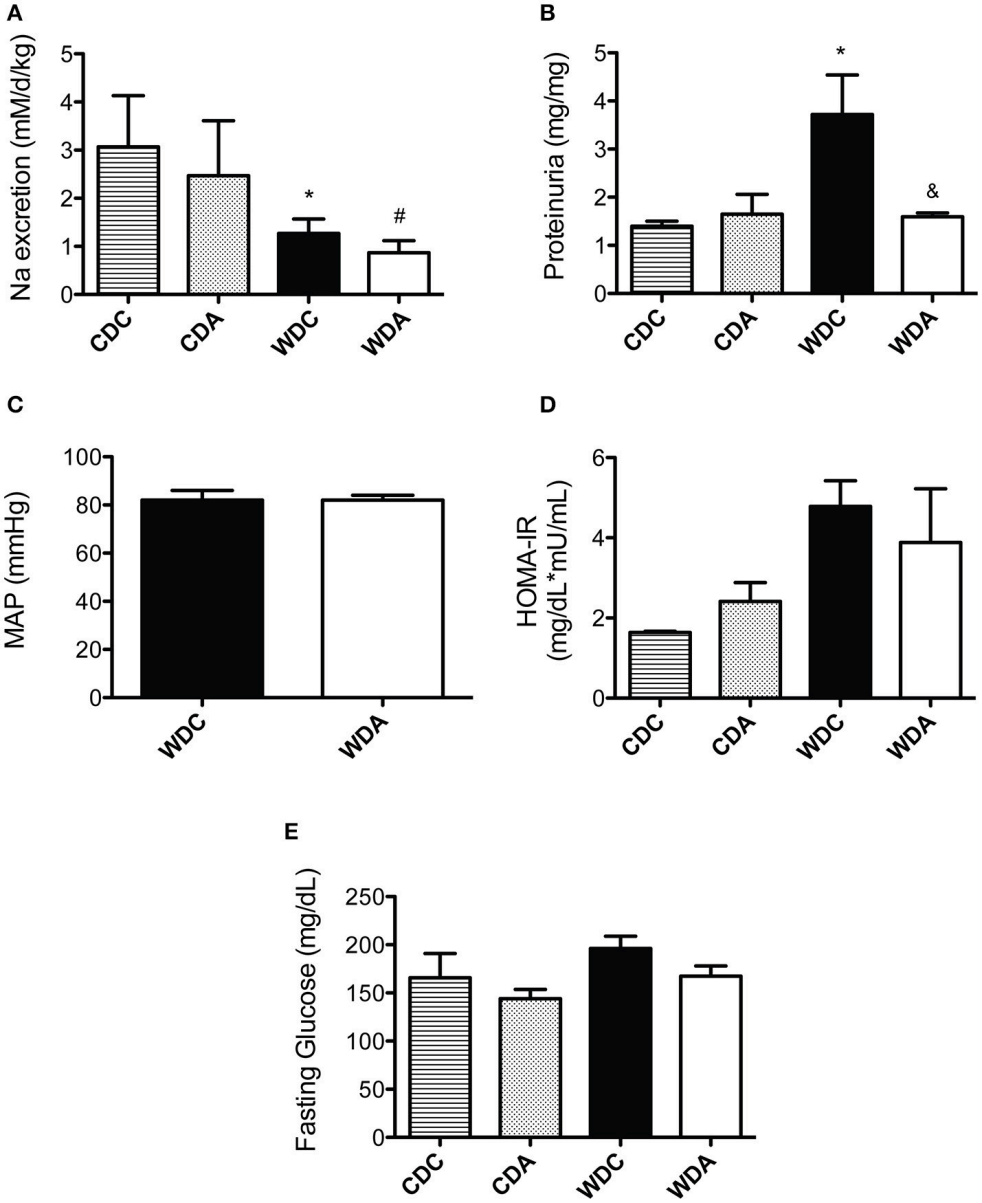
Effects of Western diet (WD) consumption and amiloride treatment on kidney function, blood pressure, and glucose metabolism. **(A)** Daily Na^+^ excretion in 23-week-old female mice fed either a control diet (CD) or a WD and treated (CDA, WDA) or not (CDC, WDC) with amiloride (1 mg/kg/day) in the drinking water. **(B)** Proteinuria expressed as Protein (mg)/Creatinine (mg) in the same mice cohorts. **(C)** Mean arterial pressure (MAP) as determined via carotid artery catheterization in WDC and WDA 24-week-old female mice. **(D)** Homeostatic model assessment of insulin resistance (HOMA-IR) in the same mice cohorts as in **(A). (E)** Fasting circulating glucose in the same mice cohorts as in **(A)**. Data are means ± SE of *n* = 3–13. ^*^P < 0.05 vs. CDC, ^#^vs. CDA, ^&^vs. WDC.

As ENaC is expressed in the collecting duct of the kidney, amiloride administration can affect kidney Na^+^ excretion and blood pressure. Therefore, we investigated the effect of very-low-dose amiloride given to mice on these two main parameters of kidney function. Urinary Na^+^ levels were reduced in mice fed a WD and were not affected by very-low-dose amiloride administration (Figure 2A). In comparison, proteinuria was increased by consumption of a WD and this increase was prevented by amiloride (Figure 2B). Because amiloride had significant kidney function effects (proteinuria) only in WD-fed mice, we measured blood pressure by Millar aortic catheter only in those animals. We found that amiloride had no significant effects on mean arterial pressure in animals fed a WD (Figure 2C). These data indicate that, at the very-low-dose we used, amiloride had no impact on natriuresis or blood pressure, but reduced proteinuria.

We have previously shown that female mice fed a WD are insulin resistant, as determined by HOMA-IR (the homeostatic model assessment of insulin resistance; Jia et al., 2015a). Therefore, we determined whether very-low-dose amiloride affected insulin sensitivity. Amiloride had no effect on blood glucose levels or HOMA-IR values (Figure 2D), which indicated very-low-dose amiloride did not affect insulin sensitivity. In addition, consistent with our previous findings, circulating levels of glucose in fasted mice were not different between any of the groups (Figure 2E).

### *In vivo* amiloride administration prevents aortic PWV increases and reduces aortic endothelial stiffness in female mice fed a WD

**Figure 3:**
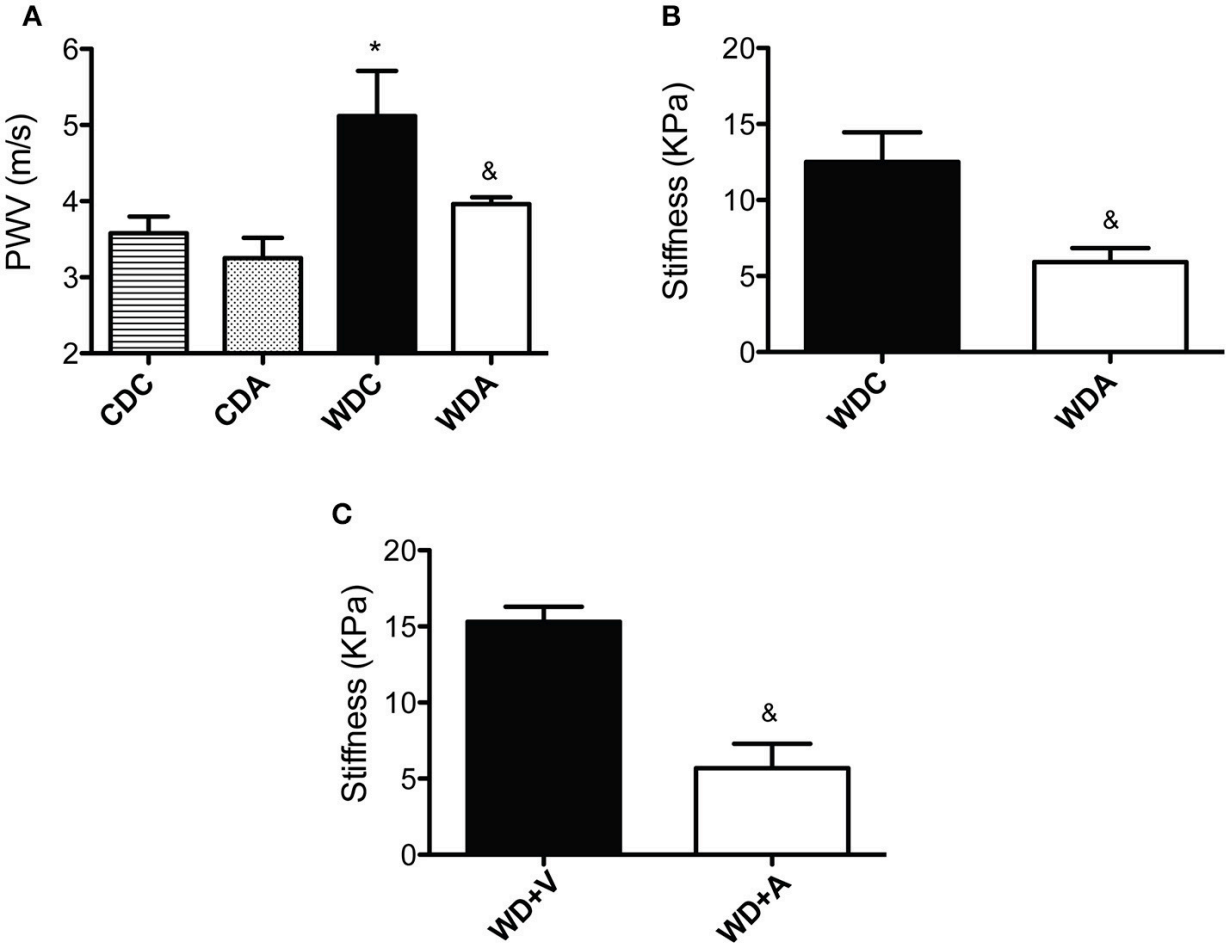
Amiloride reduces vascular stiffness. **(A)** Pulse wave velocity (PWV) in 23-week-old female mice fed either a control (CD) or a Western diet (WD) and treated (CDA, WDA) or not (CDC, WDC) with amiloride (1 mg/kg/day) in the drinking water. **(B)** Endothelial stiffness as measured by atomic force microscopy in WDC and WDA female mice. **(C)** Endothelial stiffness as measured by atomic force microscopy in female mice fed WD and treated *ex vivo* with vehicle control (WD+V) or with amiloride (WD+A). Data are means ± SE of *n* = 5–13. ^*^P < 0.05 vs. CDC, ^&^vs. WDC or WD+V.

Alterations in blood pressure are known to affect arterial stiffness and confound interpretations of PWV. Therefore, a primary goal of this study was to use a very-low-dose of amiloride that did not impact blood pressure (Jia et al., 2016) and determine whether it would prevent the development of arterial stiffening in female mice fed a WD. Previously, we have shown that WD feeding does not change arterial pressure in female mice (Jia et al., 2015b). In this investigation, we found no significant changes in blood pressure associated with amiloride treatment in WD-fed mice as shown above and as previously reported (Jia et al., 2016). Importantly, as we have also previously shown, consumption of a WD increased PWV in female mice and this increase was prevented by *in vivo* administration of amiloride (Figure 3A). In comparison, amiloride had no effect on the PWVs of mice fed a CD. This indicated that relatively selective EnNaC inhibition prevented the stiffening of the aorta induced by WD feeding in female mice without altering blood pressure or sodium excretion.

Because amiloride did not cause any changes in PWV in mice fed a CD, we concentrated our efforts on mice fed a WD as we interrogated the effects of very-low-dose amiloride on vascular characteristics *ex vivo*. We had previously shown that WD feeding increases the stiffness of aortic endothelium in female mice (Jia et al., 2016). Therefore, in order to determine if amiloride affected endothelial stiffness in mice fed a WD, we measured the deformability of the endothelium in aortic explants from female mice fed a WD with or without amiloride. *Ex vivo* measurements of stiffness using atomic force microscopy showed that amiloride reduced endothelial stiffness in the aortae of female mice fed a WD by more than 50% (Figure 3B). To determine whether these results were caused by direct actions of amiloride on the vascular wall, aortic samples from WDC mice were exposed *ex vivo* to 1 μM amiloride or vehicle control for 4 h. Similar to the results obtained from *in vivo* studies, *ex vivo* exposure to amiloride significantly reduced endothelial stiffness as compared to samples exposed to vehicle control (Figure 3C). These results suggest that amiloride softens the vascular endothelium in the aortae of female mice fed a WD through mechanisms associated with its direct actions on components of the vascular wall.

### Amiloride improves resistance artery endothelial function in female mice fed a WD

**Figure 4:**
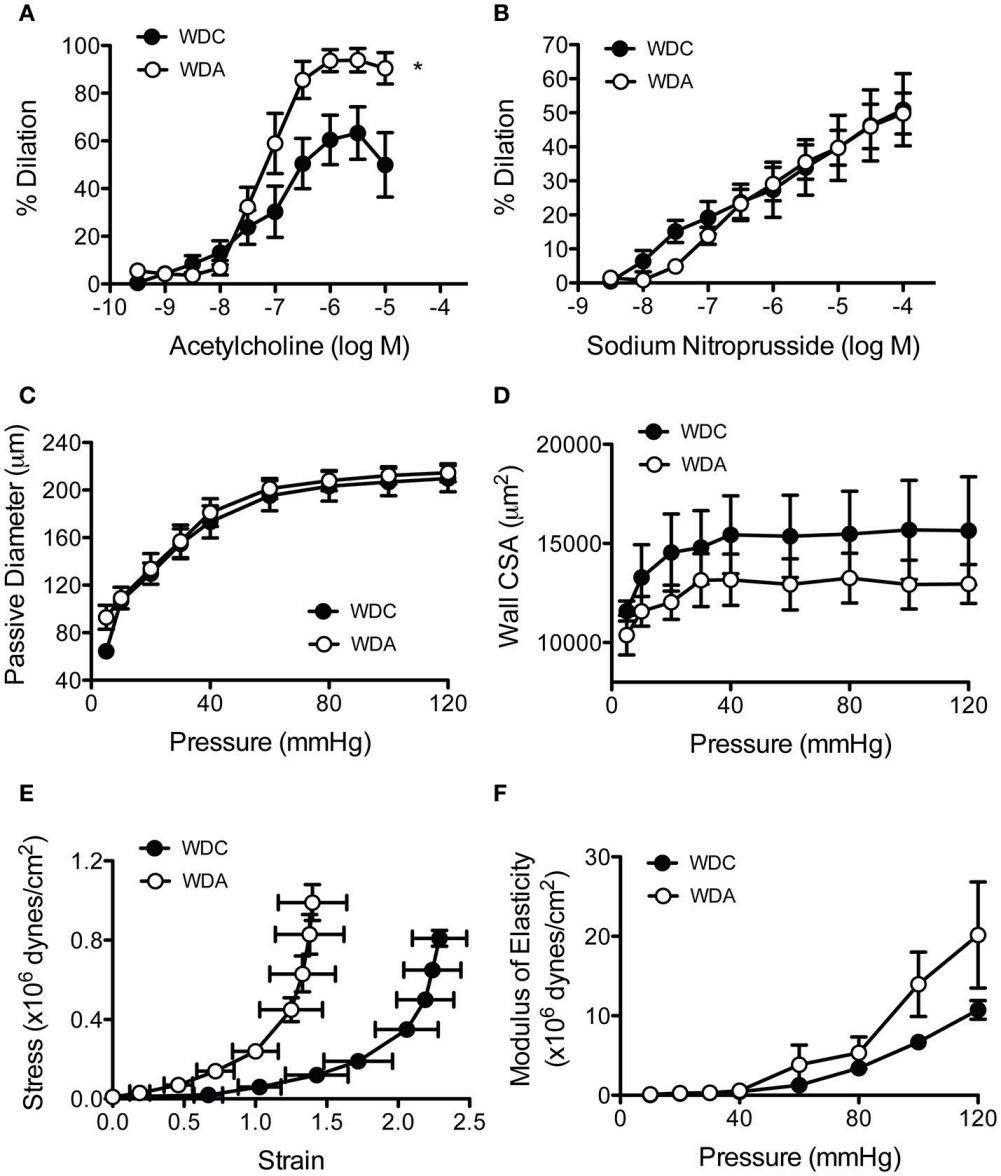
Amiloride improves endothelial function and reduces the distensibility of mesenteric arteries from female mice fed a Western diet (WD). **(A)** Percent acetylcholine-induced dilation of isolated cannulated and pressurized mesenteric arteries obtained from 24-week-old female mice fed a Western diet (WD) and treated (WDA) or not (WDC) with amiloride (1 mg/kg/day) in the drinking water. **(B)** Percent sodium nitroprusside-induced dilation of the same arteries as in **(A). (C)** Passive pressure diameter curves of the same mesenteric arteries as in **(A). (D)** Cross-sectional area (CSA) of the arterial wall from the same vessels as in **(A). (E)** Strain-stress relationships of mesenteric arteries from the same vessels as in **(A). (F)** Incremental elastic moduli of elasticity of the same mesenteric arteries as in **(A)**. Data are means ± SE of *n* = 4–5. ^*^P < 0.05 vs. WDC.

Amiloride acting through its effects on EnNaC has been shown to improve NO production in cultured endothelial cells exposed to aldosterone (Oberleithner et al., 2006), and we have previously shown very-low-dose amiloride improves endothelium-dependent vasodilatory responses of aortae from WD-fed female mice. In order to determine if these effects also occur in the microcirculation, we measured endothelium-dependent and -independent vasodilatory responses of mesenteric resistance arteries. *In vivo* administration of very-low-dose amiloride to WD-fed female mice increased the vasodilatory responses of mesenteric arteries to increasing concentrations of the endothelium-dependent vasodilator acetylcholine (Figure 4A). In comparison, amiloride had no effects on the vascular responses to increasing concentrations of the NO donor and endothelium-independent vasodilator sodium nitroprusside (Figure 4B). This indicated that inhibition of EnNaC improved endothelium-dependent vasodilatory responses in female mice fed a WD in both conduit and resistance arteries. In mesenteric resistance arteries, amiloride did not induce significant changes in vascular remodeling as assessed by measurements of maximal passive diameter (Figure 4C), but had a non-significant tendency to reduce the cross-sectional area of the arterial wall (Figure 4D). Mechanically, there was a left shift on the strain-stress relationship curves in vessels from animals receiving amiloride (Figure 4E). This occurred because vessels from mice not receiving amiloride achieved lower passive diameters at the smallest intravascular pressure tested (5 mmHg). Nevertheless, no significant changes were observed in the incremental modulus of elasticity or the overall compliance of mesenteric arteries (Figure 4F).

### Amiloride differentially affects structure and remodeling between aortae and mesenteric arteries in female mice fed a Wd

**Figure 5:**
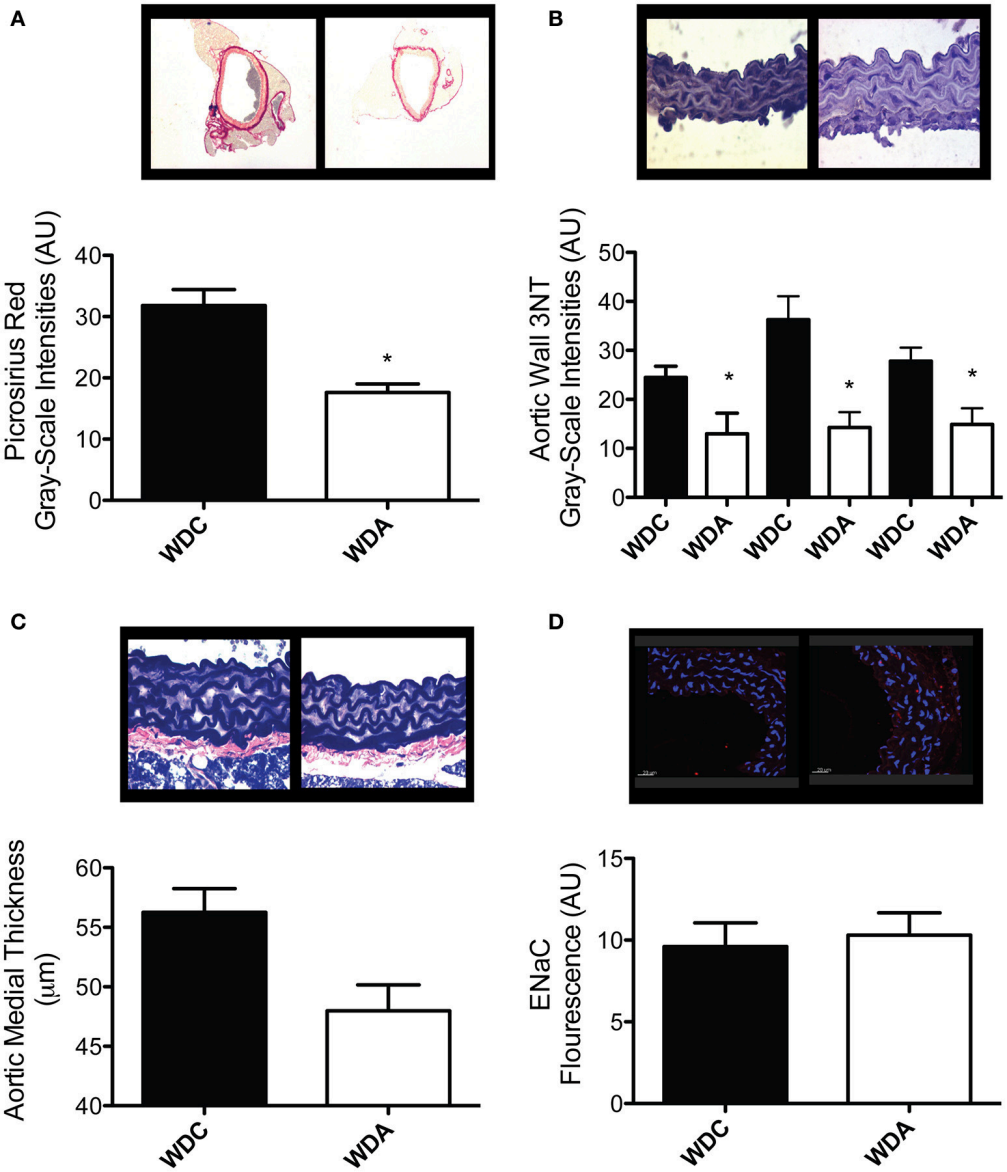
Amiloride reduces fibrosis and oxidative stress without altering EnNaC abundance in aortae of female mice fed a Western diet (WD). **(A)** Top panels are representative images (red = collagen), while the bottom panel represents the quantification of fibrosis as determined by picrosirius red staining in aortae from 24-week-old female mice fed a WD and treated (WDA) or not (WDC) with amiloride (1 mg/kg/day) in the drinking water. **(B)** Top panels are representative images (brown = 3NT), while the bottom panel represents the quantification of oxidative stress as measured by presence of 3-nitrotyrosine (3NT) in the intima (left bars), media (center bars), and adventitia (right bars) in aortae from the same mice cohorts as in **(A). (C)** Top panels are representative images (blue = elastin), while the bottom panel represents the quantification of medial thickness of aortae from the same mice cohorts as in **(A). (D)** Top panels are representative images (blue = nuclei, red = EnNaC), while the bottom panel represents the quantification of the presence of EnNaC in the endothelium of aortae from the same mice cohorts as in **(A)**. Data are means ± SE of *n* = 4–7. ^*^P < 0.05 vs. WDC.

**Figure 6:**
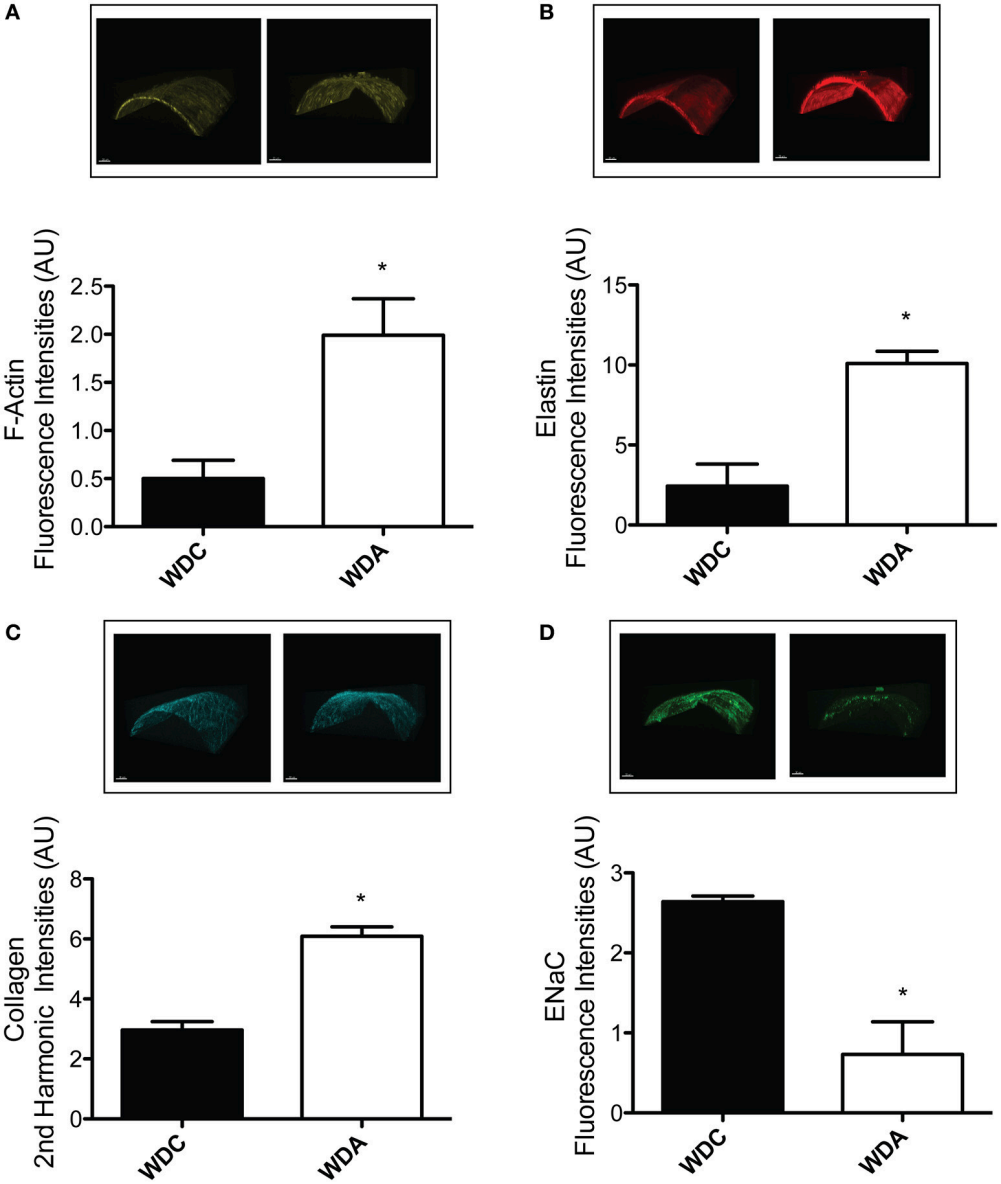
Amiloride induces the arterialization of mesenteric arteries in female mice fed a Western diet (WD). **(A)** Top panels are representative images, while the bottom panel represents the quantification of medial F-actin content in mesenteric arteries from 24-week-old female mice fed a WD and treated (WDA) or not (WDC) with amiloride (1 mg/kg/day) in the drinking water. **(B)** Top panels are representative images, while the bottom panel represents the quantification of elastin content in the same mesenteric arteries as in **(A). (C)** Top panels are representative images, while the bottom panel represents the quantification of collagen content in the same mesenteric arteries as in **(A). (D)** Top panels are representative images, while the bottom panel represents the quantification of EnNaC presence in the same mesenteric arteries as in **(A)**. Data are means ± SE of *n* = 3–4. ^*^P < 0.05 vs. WDC. All images were contrast-enhanced equally to improve their visualization in print. All analyses were performed using raw data.

**Figure 7:**
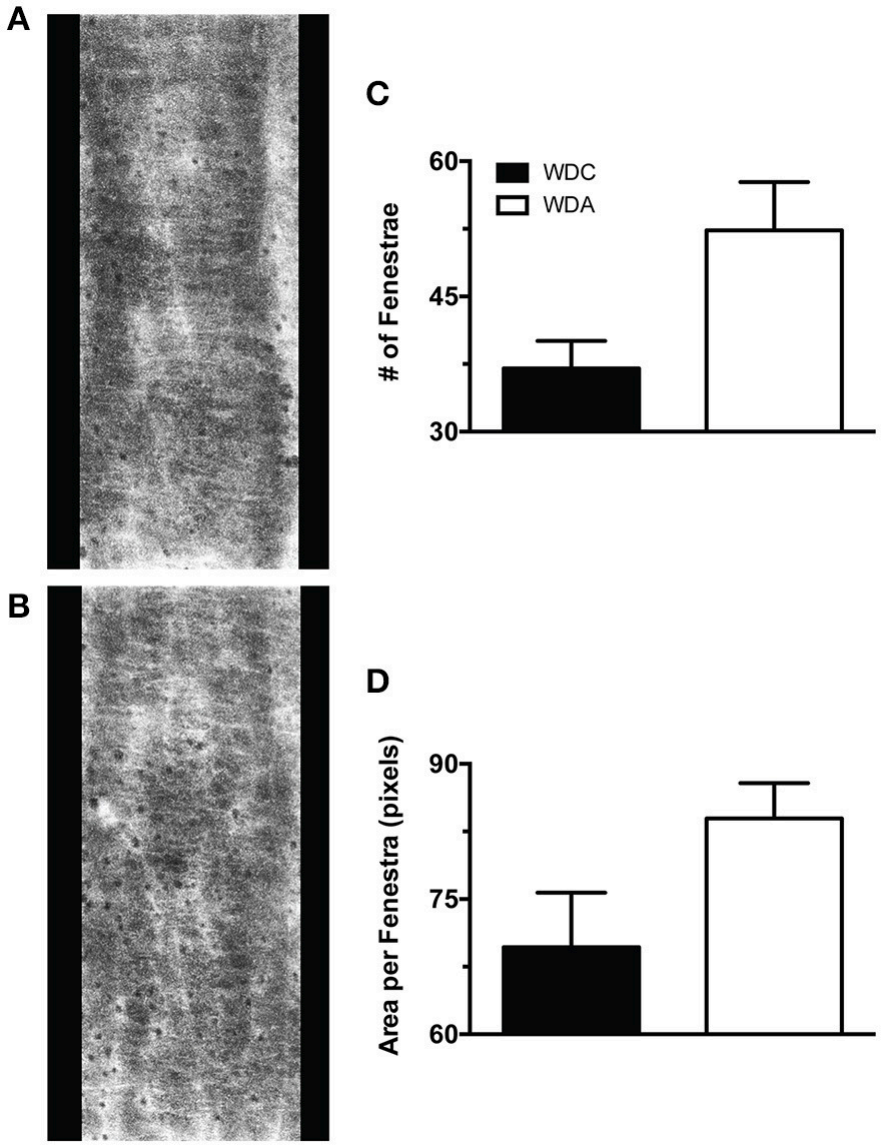
Effects of amiloride treatment on the characteristics of mesenteric artery internal elastic lamina fenestrae in Western diet (WD) fed female mice. **(A)** Representative image of the internal elastic lamina and its fenestrae of a mesenteric artery obtained from a 24-week-old female mouse fed a WD and not treated with amiloride. **(B)** Representative image of the internal elastic lamina and its fenestrae of a mesenteric artery obtained from a 24-week-old female mouse fed a WD and treated with amiloride. **(C)** Number of fenestrae per area (400 × 1,024 pixels) in the internal elastic lamina of 24-week-old female mice fed a WD and treated or not (WDA, WDC, respectively) with amiloride (1 mg/kg/day) in the drinking water. **(D)** Mean area per fenestra in the same vessels as in **(C)**. Data are means ± SE of *n* = 3–4. All images were contrast-enhanced equally to improve their visualization in print. All analyses were performed using raw data.

To determine the effects of *in vivo* very-low-dose amiloride administration on aortic structure, we performed histological studies in aortic explants from female mice fed a WD with or without amiloride. Picrosirius red staining indicated that amiloride reduced the amount of fibrillar collagen in aortae of female mice fed a WD (Figure 5A). Amiloride also reduced the staining associated with presence of nitrotyrosine in all layers of the aortic wall, including the intima, media, and adventitia (Figure 5B). Amiloride did not reduce nitrotyrosine in perivascular adipose tissue (data not shown), nor did it alter the amount of EnNaC present in aortic endothelium (Figure 5D). Structurally, there was a slight but not significant (*P* = 0.06) reduction of aortic medial thickness in animals treated with amiloride (Figure 5C). In mesenteric arteries, amiloride administration was associated with a significant increase in filamentous (F)-actin in the medial layer of the vessels and an overall increase in elastin and fibrillar collagen content in the vascular wall (Figures 6A–C). In contrast to aortae, amiloride reduced the amount of EnNaC present in the mesenteric arteries obtained from female mice fed a WD (Figure 6D). Although, we observed an increase in vascular elastin content, there was only a tendency for an increase in the size and amount of fenestrae present in the internal elastic lamina of the arteries (Figure 7). Overall, these results suggest that amiloride administration at a dose not affecting Na^+^ excretion or blood pressure, prevents WD-induced fibrosis and oxidative stress in the aorta, while it increases the presence of smooth muscle F-actin and extracellular matrices in mesenteric resistance arteries.

## Discussion

The primary finding of the present investigation is that very-low-dose amiloride prevented the development of aortic stiffening, resistance artery endothelial dysfunction, and proteinuria in young female mice fed a WD. This finding has important translational implications. First, obese women have a greater incidence of arterial stiffening and an increased risk for CVD and CKD than men (Ryden Ahlgren et al., 1995; Zebekakis et al., 2005), which makes our results highly relevant to this human population. Second, the WD, high in fat and refined sugars, used in our experiments is highly representative of the diet consumed today in Western societies, as compared to other diet-obesity models in which only fat content in the diet is increased. Third, our results indicate that a very-low-dose of the ENaC inhibitor, amiloride, a currently prescribed pharmaceutical drug, could be of protective use at doses not affecting blood pressure to prevent vascular endothelial dysfunction and arterial stiffening in females with diet-induced obesity. Therefore, our results imply that low doses of amiloride could be used to reduce overall CVD in obese women.

In this investigation, we initially examined the effects of very-low-dose amiloride on body composition, glucose metabolism, blood pressure, renal Na^+^ excretion, and proteinuria. Our results indicate that 1 mg/kg/day amiloride did not alter body composition, insulin sensitivity or blood pressure in obese female mice fed a WD. Neither did amiloride alter kidney Na^+^ excretion at the steady state of 5 months on WD feeding suggesting no effect on kidney ENaC-mediated Na^+^ reabsorption. Our measurements of Na^+^ balance in WD-fed female mice have also shown no alteration in Na^+^ intake (data not shown). In contrast, very-low-dose amiloride prevented the increase in proteinuria associated with consumption of a WD. There is increasing evidence which suggests that vascular stiffness predicts CKD and, in this context, measures of proteinuria are a marker of kidney function closely linked to vascular stiffness (Liu et al., 2010). As there were no increases in blood pressure in this model, the elevated proteinuria in WD-fed mice may be reflective of glomerular endothelial injury associated with alterations in vascular stiffness over time and thereby a functional marker for the improvements in vascular reactivity and structure observed in this study. Previous work in humans support the notion that blood pressure elevations do not need to occur in order for blood vessels to stiffen, especially as it relates to the development of proteinuria (Schultz et al., 2001; Liu et al., 2010). Alternatively, very-low-dose amiloride could have prevented proteinuria via its ENaC-independent effects on urokinase-type plasminogen activator, which are known to reduce proteinuria (Zhang et al., 2012). However, amiloride doses known to attenuate plasminogen activation also reduce blood pressure (Oxlund et al., 2014). Therefore, whether very-low-dose amiloride improved proteinuria via effects on renal vascular function and structure, or via its effects on plasminogen activation remains to be determined.

As amiloride did not affect body composition, blood pressure, or insulin sensitivity in female mice fed a WD, our major finding that amiloride reduced aortic PWV in those mice suggests amiloride reduced vascular stiffness via direct actions on the vascular wall. It has been shown that the expression and activity of EnNaC increases cellular stiffness and reduces the capacity of endothelial cells to produce NO (Oberleithner et al., 2007). This is supported by our previous findings that female mice fed a WD had reduced aortic vasodilatory responses to the endothelium NO-dependent vasodilator acetylcholine, which occurred concomitantly with an increased presence of aortic EnNaC (Jia et al., 2016). Therefore, in our present investigation, amiloride most likely prevented WD-induced aortic stiffness via preventing EnNaC activation. Amiloride inhibits EnNaC activity by reducing the cell-membrane expression of the channel and by lowering its conductance for Na^+^ ions. We found that the endothelial specific presence of ENaC was unaltered by amiloride in the aortae of female mice fed a WD. Therefore, it is likely that amiloride decreased EnNaC activity in the aorta without altering its expression. In this regard, 20 weeks of treatment with very-low-dose amiloride may have been insufficient to induce changes in aortic protein expression, or it is possible that this extended treatment period may have resulted in a compensatory upregulation of EnNaC in this conduit artery. Comparatively, we found that EnNaC presence was reduced in mesenteric arteries from WD-fed mice treated with amiloride. This finding is in line with previous data showing that acute treatment with amiloride reduced EnNaC expression in human umbilical vein endothelial cells (Kusche-Vihrog et al., 2008) and that *ex vivo* exposure of arteries explanted from human subjects to amiloride softened their endothelial surface (Lenders et al., 2015). However, in the latter study, softening of the endothelium by *ex vivo* exposure to amiloride did not occur when subjects had significantly increased PWVs (Lenders et al., 2015). Therefore, an additional potential scenario is that endothelial cells in the aorta vs. those in mesenteric arteries react differently to amiloride, perhaps due to their exposure to different levels of shear stress, which are known to affect endothelial cell physiology (Chistiakov et al., 2017). Nevertheless, our findings are consistent with amiloride exerting an overall inhibitory effect on WD-induced increases in EnNaC expression and function (Kusche-Vihrog et al., 2008; Hanukoglu and Hanukoglu, 2016). Importantly, our results indicating that amiloride reduced PWV in female mice fed a WD were accompanied by a reduction in endothelial stiffness, as measured in aortic explants using atomic force microscopy. Moreover, in order to corroborate that a direct effect of amiloride on the vasculature could be responsible for reducing vascular stiffness, we treated aortic explants from female mice fed a WD with amiloride *ex vivo*. Results indicate that amiloride acting directly on vascular tissues is able to reduce endothelial stiffness, which further suggests that our *in vivo* treatment with amiloride likely reduced aortic stiffness in females consuming a WD by acting directly on EnNaC activity.

As mentioned above, increases in EnNaC activity reduce the capacity of vascular endothelium to produce NO (Oberleithner et al., 2007). Therefore, our previous observation that amiloride increased the endothelium-dependent vasodilatory capacity of aortae (Jia et al., 2016) and our current finding that it also improved it in mesenteric arteries suggests that amiloride improved the production of endothelial NO by reducing the activity of EnNaC. Amiloride also prevented WD-induced increases in aortic oxidative stress, as determined by the reduced presence of nitrotyrosine in the vascular wall. Reactive oxygen species are known to increase the open probability of ENaC (Ma, 2011). Therefore, an increase in Na^+^ entry into endothelial cells in response to increased reactive oxygen species and EnNaC activity should increase the stiffness of the endothelium and reduce its capacity to produce NO (Oberleithner et al., 2007). This is consistent with our finding that amiloride reduced aortic oxidative stress in conjunction with reduced endothelial stiffness. Furthermore, NO is also known to modulate collagen deposition and fibrosis through multiple pathways, which include direct and indirect managing of collagen synthesis and augmented collagen degradation via activation of matrix metalloproteinases (Kolpakov et al., 1995; Simmers et al., 2015; Song et al., 2016). NO is also a major inhibitor of tissue-type transglutaminase, an enzyme that crosslinks collagen and reduces its degradation (Jandu et al., 2013). Therefore, our finding that amiloride reduced aortic fibrosis in female mice fed a WD is consistent with a scenario in which amiloride allowed for increased production and bioavailability of endothelial NO, and consequently a reduction in aortic fibrosis and stiffness via modulation of collagen synthesis, crosslinking and degradation.

In mesenteric arteries, the increased endothelium-dependent vasodilatory capacity induced by amiloride was associated with a reduced presence of EnNaC, but not with a reduction in vascular stiffness. Contrary to expectations, amiloride reduced the distensibility of mesenteric arteries obtained from female mice fed a WD. However, this reduced distensibility was associated with a diminished capacity of arteries to have smaller diameters at the lowest pressure tested, and not with a reduced maximal passive diameter at high pressures. This, in conjunction with the significant increase in F-actin, elastin, and collagen content in the wall of these mesenteric arteries, suggest that amiloride through its capacity to increase NO bioavailability favored the arterialization of these resistance vessels. Indeed, it has been previously shown that NO promotes the differentiation and maintenance of a vascular smooth muscle cell contractile phenotype with an increase in F-actin content and the overall arterialization of blood vessels, which specifically includes augmented extracellular matrix expression by differentiated vascular smooth muscle cells (Lincoln et al., 2006; Simmers et al., 2015). Interestingly, although there was an increased amount of elastin present in the internal elastic lamina of resistance arteries from amiloride treated mice, there was also a tendency for the fenestrae present in the lamina to be more abundant and have bigger sizes. Fenestrae in the internal elastic lamina are known to be avenues of communication between the endothelium and vascular smooth muscle that allow for a more expedited movement of molecules such as NO (Straub et al., 2012). Whether the non-significant increase in fenestrae number and size we observed in resistance arteries from mice treated with amiloride increased NO diffusion from the intima to the medial layer of the vessel remains to be determined. Nonetheless, a differential effect of increased NO bioavailability in aortic vs. mesenteric resistance artery smooth muscle cells may be responsible for the apparently divergent effects of amiloride on aortic and mesenteric resistance artery stiffness.

**Figure 8:**
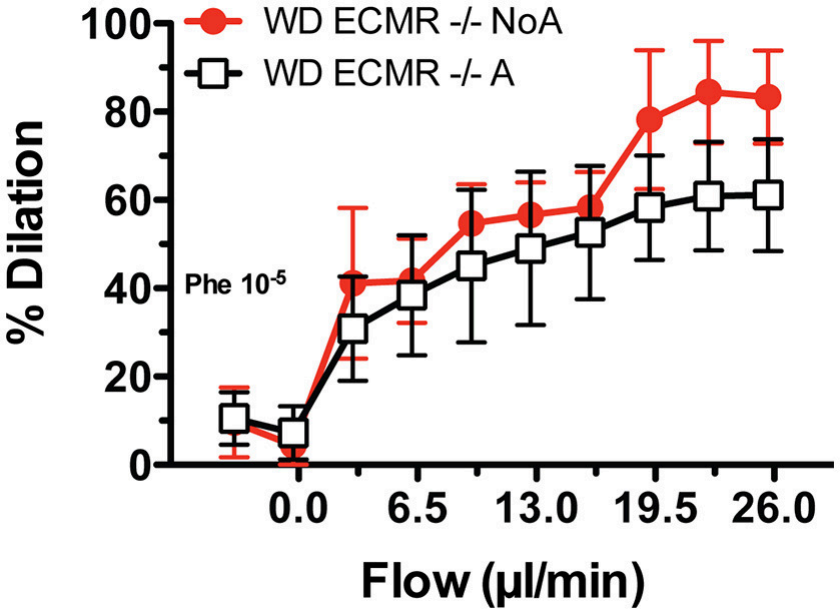
*Ex vivo* exposure to amiloride does not increase the vasodilatory responses to flow of mesenteric arteries obtained from endothelium-specific mineralocorticoid receptor knock out female mice (ECMR^−/−^). Isolated cannulated and pressurized mesenteric arteries from ECMR^−/−^ female mice fed a Western diet (WD) were incubated with amiloride (1 uM) for 20 min, pre-constricted with 10^−5^ M phenylephrine (Phe) and then exposed to increasing levels of intraluminal flow while maintaining mean intravascular pressure at 70 mmHg in order to determine flow-mediated vasodilatory effects. The vasodilatory effects of flow on ECMR^−/−^ arteries treated with vehicle control (NoA) that were published previously (red line)^3^ are included for comparison. Data are means ± SE of *n* = 4–5.

The major findings reported herein support our overarching hypothesis that aortic stiffening in females consuming a WD depends on activation of MRs in endothelial cells that in turn enhance the presence and activity of EnNaC to promote endothelial stiffness and dysfunction. Consequently, our specific hypothesis tested here was that reducing the activity of EnNaC with very-low-dose amiloride would decrease arterial stiffness in female mice fed a WD, and that this arterial de-stiffening would be accompanied by amelioration of arterial fibrosis, endothelial dysfunction, oxidative stress, and endothelial stiffening, which our results substantiate. Previously, we showed that isolated mesenteric arteries from wild type female mice fed a WD, which have increased vascular MR expression (Jia et al., 2016), also have improved flow-induced vasodilatory responses when exposed *ex vivo* to amiloride (Jia et al., 2016). Herein, we exposed isolated mesenteric arteries from ECMR^−/−^ female mice fed a WD to amiloride *ex vivo*, and found that exposure to amiloride did not improve flow-induced vasodilation (Figure 8). This further supports our overarching hypothesis that consumption of a WD and obesity in females is associated with increased MR signaling (Cooper et al., 2012), which is known to increase expression and translocation of ENaC to the cell surface (McEneaney et al., 2008). ENaC is constitutively active and entrance of Na^+^ through EnNaC increases cytoskeletal actin fibers (Mazzochi et al., 2006; Kusche-Vihrog et al., 2014). The resultant endothelial cortical stiffening reduces eNOS activation and endothelial NO bioavailability (Oberleithner et al., 2007), which in turn promotes arterial stiffening and an increased risk for CVD and CKD. Specific details on the mechanisms associated with this pathway remain to be fully elucidated. However, our current results clearly indicate that therapeutic intervention on this pathway at the level of EnNaC activity with very-low-dose amiloride is sufficient to ameliorate the pathological effects of WD-induced obesity on the vasculature without alterations in blood pressure or natriuresis. Further studies will be required to translate these findings to clinical practice.

## Ethics statement

This study was carried out in accordance with the recommendations of the guidelines from the National Institutes of Health. The protocol was approved by the animal care and use committees at the University of Missouri and the Harry S. Truman Veterans' Memorial Hospital.

## Author contributions

LM and JS conceived and designed the study, interpreted data, and edited the manuscript. AA, FR, GJ, JH, VD, and BB performed experiments, made figures, and analyzed data. LM, AW, and RN interpreted data and wrote and edited the manuscript. All authors approved the final version of the manuscript.

### Conflict of interest statement

The authors declare that the research was conducted in the absence of any commercial or financial relationships that could be construed as a potential conflict of interest.
